# State Care of Consumptives

**Published:** 1898-03

**Authors:** 


### STATE CARE OF CONSUMPTIVES.

AT THE recent meeting of the medical society of the state of New York, Dr. John H. Pryor, of Buffalo, presented a paper on The state and county care of the consumptive. After citing statistics showing the great mortality from consumption in this state, the effects of attempts at prevention were considered and the conclusion reached that while the number of deaths in proportion to the population decreased, the percentage of deaths from the disease seems to increase. About 13,000 of the population die from consumption in this state, or about 11 per cent. of all deaths are due to that cause.

The necessity for the proper care of incurable cases in special hospitals, with a view to segregation and better opportunity for improvement, was urged as a humane and sanitary measure. These methods of attacking consumption, if thoroughly employed, are scientific and promising, but in the opinion of the author were insufficient in that no provision is made for curable cases. The plan advised is experimental care of the incipient consumptive by the state, through the establishment of a colony for consumptives during the curable stage of the disease in the Adirondacks, on the state reserve. It can no longer be questioned that from 25 to 50 per cent. of incipient consumptives may recover under proper climatic and hygienic conditions. The Adirondack region furnishes the best health resort for this purpose east of Colorado and the state owns an enormous tract of land which could not be put to better use. While the well-to-do may be able to seek the only relief known for this disease, the vast majority of consumptives are denied this opportunity. Those in moderate circumstances or the indigent sick must struggle until all hope is lost and they receive aid when too late to be of any real benefit.

It is an admitted fact that thousands of consumptives die annually in this state because they are too poor to avail themselves of the advantages proposed by Dr. Pryor. There is little excuse at present for neglecting to make an early diagnosis and the chance could be given to many to get well and be useful citizens. We seem to be working at the wrong end of a problem and, as Dr. Pryor says, the care of the consumptive is an anomaly in our system of charities. The indigent sick when suffering from any other disease are given the best medical treatment, but the consumptive whose only chance for recovery must be provided by changed climatic conditions is entirely lost sight of. The cities and counties expend large sums of money for the relief of the patient and his family after any hope for his recovery has vanished. Until then he is neglected and the increasing knowledge of the infectiousness of the disease makes his life and efforts to gain support more and more difficult.

Probably the greatest affliction which a desirable citizen suffers from today is consumption, because nature affords him a chance to recover near his home, but the expense is prohibitive in most cases. It would be much wiser to spend the money where it will bring a return than to employ it to comfort the hopeless sufferer. Why not support the consumptive until he is well and give him treatment where there is some promise of a good result? The purpose of such a colony should not be to care for the poor alone, but those who can pay all or a part of the cost should be given this necessary opportunity. There is no reason why a colony for consumptives should not be made partially self-supporting. After the period of rest has elapsed some of the patients, perhaps, might be placed upon light duty of some kind to avoid the dangers of pauperism. To be at all successful only incipient cases should be admitted and the results will depend largely upon the care used in selecting cases. Unless developed slowly and cautiously the expense would be great.

The strongest humane argument in favor of the plan is the strongest economical one against it. It is so much needed and the demand will be so great for relief that the cost might be enormous. At any rate the great scourge of tuberculosis must be attacked by the state in some way, at some time, and the scheme seems to be rational and feasible. We know of no other method which has received such widespread support and commendation. The Charlotte (N.C.) *Medical Journal*, for October, 1896, makes editorial reference to this subject as follows: "The state should erect sanitariums in mountainous districts and give the poor the same climatic advantages enjoyed by the rich." The article referred to we understand is from the pen of Dr. Julius Ullman, of this city. The only objection seems to be the fear of another large expense added to the state and the creation of a new institution to care for a new class of dependents. Careful consideration of the philanthropic side of the matter should remove this hesitancy. A small colony could be started on the cottage plan and time could be trusted to show the necessity for growth. The expense could be controlled and wisely increased by those who have the power to make appropriations.

It is probable that the legislature will appoint a committee to investigate the suggestions and plans presented at the Albany meeting, and it is to be hoped that the importance of the matter will lead to a thorough examination of the means for lessening the number of consumptives in this state.

---

UNDER the head of society proceedings in this issue we publish an abstract of Dr. Kelly's paper read at the last annual meeting of the Southern Surgical and Gynecological Association, relating to the sources and diagnosis of pyurias. Dr. McMurtry's discussion on the subject is published in full, which we think will make instructive reading for those interested in this important subject. So many false claims have been made in society discussions and medical journals for the much-vaunted ureteral catheterisation for both diagnosis and treatment, that it is highly satisfactory to have the subject set forth to the profession in its true light.

---

THE appalling disaster that befell the United States battleship Maine, in Havana harbor, February 15, 1898, is a subject of comment all over the world, and has brought messages of sympathy to the government at Washington from almost every civilised ruler. The great loss of life and the large number of maimed present the most paramount questions connected with this explosion. The value of the ship, though great, is of secondary moment. It is gratifying that the medical officers escaped injury, thus enabling them to succor the wounded and contribute to their immediate relief. We make reference to this subject here, and at this time, to express our sympathy with the medical staff of the navy and especially to surgeon Heneberger and his assistants for the trying position in which they are placed. The destruction of the Maine, when peacefully riding at anchor in smooth water, is a reminder that at no time in peace or war is service in the naval medical staff a sinecure or place of safety.

